# Comparative Phylogeography in Fijian Coral Reef Fishes: A Multi-Taxa Approach towards Marine Reserve Design

**DOI:** 10.1371/journal.pone.0047710

**Published:** 2012-10-30

**Authors:** Joshua A. Drew, Paul H. Barber

**Affiliations:** 1 Department of Ecology, Evolution and Environmental Biology, Columbia University, New York, New York, United States of America; 2 Department of Ecology and Evolutionary Biology, University of California Los Angeles, Los Angeles, California, United States of America; University of Texas, United States of America

## Abstract

Delineating barriers to connectivity is important in marine reserve design as they describe the strength and number of connections among a reserve's constituent parts, and ultimately help characterize the resilience of the system to perturbations at each node. Here we demonstrate the utility of multi-taxa phylogeography in the design of a system of marine protected areas within Fiji. Gathering mtDNA control region data from five species of coral reef fish in five genera and two families, we find a range of population structure patterns, from those experiencing little (*Chrysiptera talboti, Halichoeres hortulanus*, and *Pomacentrus maafu*), to moderate (*Amphiprion barberi*, Φ_st_ = 0.14 and *Amblyglyphidodon orbicularis* Φ_st_ = 0.05) barriers to dispersal. Furthermore estimates of gene flow over ecological time scales suggest species-specific, asymmetric migration among the regions within Fiji. The diversity among species-specific results underscores the limitations of generalizing from single-taxon studies, including the inability to differentiate between a species-specific result and a replication of concordant phylogeographic patterns, and suggests that greater taxonomic coverage results in greater resolution of community dynamics within Fiji. Our results indicate that the Fijian reefs should not be managed as a single unit, and that closely related species can express dramatically different levels of population connectivity.

## Introduction

Historically, marine reserves have been established using a wide variety of criteria [1], [2], yet the metrics of reserve design have not always included biological justification [3], [4]. Having an explicit scientific evaluation of potential sites for a marine reserve network reduces uncertainty in placement, and provides more effective allocation of finite conservation resources [5]. Providing a scientific justification for reserve placement also eases the adoption of the network in a policy framework because the screening process produces quantifiable (and ultimately more defensible) justifications for the reserve orientation [6].

An important factor in setting up reserve networks is quantifying the extent over which individual nodes are connected [7]. In marine systems this connectivity is most commonly brought about through larval dispersal [8], [9]. Reserve networks that are richly connected with strong links among nodes are more resilient to perturbation [10], [11], augment local fisheries by exporting larvae and, at limited spatial scales, through export of adults [12], [13]. Furthermore, placing reserves in a network buffers against uncertainty in the placement of individual reserves [14], [15].

Despite its importance to effective marine reserve design, the quantification of larval exchange is problematic. Tracking of individual late-stage larvae via direct observation is possible for some species [16], however following an individual larva from hatching to settlement in the wild is presently impossible for most species. While direct methods such as biochemical mark and recapture are feasible under some biological and oceanographic conditions [17], they are logistically intensive, and both biologically and spatially limited. Additionally, these methods are also subject to small-scale geographic, or seasonal variances in recruitment that may not be informative over larger spatial or temporal scales [18], [19]. Therefore we must use surrogates in order to understand and quantify the degree to which individual reserves are connected.

Many studies use genetic similarity as a proxy for connectivity (see [20], [21], [22], [23] for reviews). Although molecular tools suffer difficulty in evaluating connectivity on ecological time scales relevant to marine reserve design [24], the use of rapidly evolving genetic markers does allows for a more smoothed temporal scale, and can present a long-term average as opposed to a short-term snapshot measurement of dispersal [21]. Genetic techniques are particularly useful in helping identifying barriers to connectivity, since relatively few migrants per generation can reduce genetic heterogeneity [24]. Therefore, the presence of genetic structure shows the absence both of evolutionary and ecologically meaningful connectivity.

Although discerning population connectivity is important for all marine habitats, it is a particular concern for small archipelagic counties of the Pacific. This concern arises from the physical isolation of these archipelagos from potential source habitats as well as the geographic independence of the islands within the archipelagos. Previous studies that have assessed gene flow among reef fish population within Pacific Islands archipelagos have found substantial exchange within island groups [25]–[29], with only a few exceptions [30], [31]. These studies have provided important information regarding the evolution and conservation of marine biodiversity of Pacific archipelagos, however their applicability towards setting conservation priorities for reef communities as a whole is limited by their reliance on inferences from a single representative species.

**Figure 1:**
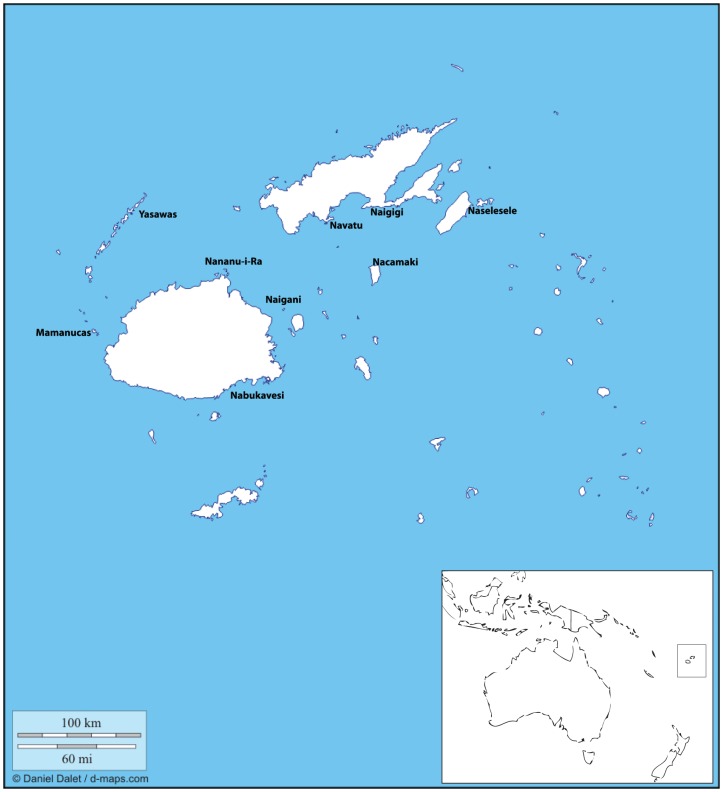
Map of the Republic of Fiji with sampling areas indicated.

The Republic of Fiji consists of over 400 islands situated in the southwest tropical Pacific (Fig. 1). These islands possess abundant coral reefs and major islands are separated by deep ocean. In 2003 the government of Fiji initiated a feasibility study for a system of marine reserves, and concluded that between 20 and 30% of their waters should be set aside in traditionally managed no-take protected areas [32]. However the exact placement of these reserves was left unresolved until a thorough scientific examination could take place. In order to provide biologically justifiable recommendations to the government we tested two hypotheses regarding the connectivity among Fijian reefs. First, we wanted to examine the geographic scale over which genetic variation can take place. Although population differentiation over small spatial scales (<100 s of Km) has been recorded in marine systems [33]–[35], differentiation within Pacific fishes at the archipelago scale is rare. We also wanted to specifically test whether the Bligh Waters, a fast flowing current which runs from east to west separating the two main islands Vanua Levu and Viti Levu, were acting as a phylogeographic barrier similar to those brought about by strong currents in Indonesia [36] and the Caribbean [37]. In the latter systems genetic differentiation among populations on either side of the flow is propagated because larvae are advected out of the system before settlement.

To examine the spatial scale of genetic differentiation and specifically whether the Bligh Waters act as a phylogeographic barrier we used data from five species of conspicuous and ecologically diverse reef fish. By choosing multiple species we are able to differentiate between concurrent phylogeographic patterns and species-specific idiosyncrasies, thus providing more biologically justifiable management recommendations.

We examined the population structure of five common fish species: *Amblyglyphidodon orbicularis*, *Amphiprion barberi*, *Chrysiptera talboti*, *Pomacentrus maafu* [Pomacentridae] and *Halichoeres hortulanus* [Labridae]. Of these, *Amb. orbicularis*, *A. barberi* and *P. maafu*, are Southwest Pacific endemics [38]–[40], while a previous study identified Fijian population of *C. talboti* as a genetically distinct lineage in Fiji [41]. In contrast *H. hortulanus* is widespread, ranging from French Polynesia to East Africa, and shows little genetic divergence among Pacific populations (unpublished).

## Materials and Methods

### In situ collection

To evaluate the comparative phylogeography of these five species as well as to test the effectiveness of the Bligh Waters as a potential barrier to dispersal we sampled across four regions: the Western Islands which lie downstream of the Bligh Waters (Mamanucas, Yasawas) the islands of Viti Levu (Nabukavesi, Nananu-i-ra, Naigani) and Vanua Levu (Navatu, Naigigi), which lie south and north of the Bligh Waters respectively and the Eastern Islands which lie upstream of the waters (Naselesele on the island of Taveuni and Nacamaki on the island of Koro, Fig. 1).

### Ethics Statement

Samples were collected under the auspices of the animal care permits of Boston University (where both authors were affiliated at the time). Permits were provided by the Fijian Ministry of Fisheries and with the knowledge and permission of the traditional reef owners, and in most cases were taken from small patch reefs just offshore from the villages.

The fish were collected using nets and spears and therefore only targeted the specific species and the specific number of individuals that were needed. Fish were collected in as humane as possible fashion, with death by spear being instantaneous, or when collected in nets using clove oil as an anesthetic.

Because of the targeted method of colleting, the physical disturbance to the reef structure was minimal. Additionally, we chose to do this study with small, abundant, and highly fecund species to minimize the environmental, ecological and fisheries impact of our research. No live fish were used in the study.

### Laboratory analysis

After collection, samples were stored *in situ* in 95% EtOH. Genomic DNA was extracted using a 10% Chelex solution [42]. A fragment of the mitochondrial DNA control region was amplified using the primers CRA and CRE [43] and sequenced using the methods outlined previously [41].

To determine the degree of genetic variation explained by regional structure we conducted an analysis of molecular variance and population fixation indices (Φ_st_ the mtDNA equivalent of F_st_) using ARLEQUIN 3.5 [44] with 1000 replications to estimate significance. Initially each population was treated as a separate entity except for *A. barberi* where small sample sizes (<7 individuals in some cases) would have made results unreliable. When no significant differences were detected within regions (defined above) the data were reanalyzed with data pooled into regional assemblages.

To estimate migration among the four regions in a coalescent framework we used the program MIGRATE 3.1.6 [45]. Data were pooled as above to test the hypothesis of regional differentiation. We used a Bayesian framework running between 500,000 and 1,000,000 generations with an initial 25% burn in. We ran two independent runs and summarized over both of them, and each run utilized the adaptive heating feature to optimize the ability of the chains to explore parameter space. Because migration estimates are susceptible to the influence of recent demographic history [46], we also calculated Tajima's D in ARLEQUIN [44] to test for a signal of demographic stability or expansion on the pooled regional data.

Additionally, to determine if any phylogeographic pattern observed was a function of the spatial scale of the Fijian Islands or due to a phylogeographic barrier, we conducted an Isolation by Distance analysis between genetic distance (pair wise Φ_st_) and geographic distance (shortest shore-line distance estimated via Google Earth) using IBD 3.15 [47]. This analysis was conducted using the individual sampling sites as opposed to the grouped regional data set in order to increase geographic resolution, unfortunately this precluded an analysis of *A. barberi* due to small sample sizes at the locality level. Significance was determined over 1000 replicates were run, with all negative genetic distances being set to zero.

## Results

We generated 544 sequences from five species ranging in length from 364 to 398 bases (Table 1, 2). Sample sizes for species ranged from 65 for *A. barberi* to 148 for *P. maafu* (Table 1). The molecular data were diverse, with all species having high haplotype diversity; the population parameter, Θ_s_ ranged from 3.44 in *P. maafu* to 34.37 in *C. talboti*, while nucleotide diversity (π) ranged from 0.01 in *P. maafu* to 0.04 in *Amb. orbicularis*. We have deposited sequences into GenBank (JX486914–JX487152, and JX506313–JX506412).

**Table 1:** [Sec s3]Results of sequencing the mitochondrial Control Region, asterisks refer to larval estimations from most related congener for which data were available (Victor 1986; Wellington & Victor 1989; Leis & Carson-Ewart 2003).

Species	Individuals	Haplotypes	π	θs (SD)	Sequence Length	Larval Duration
*Amblyglyphidodon orbicularis*	102	88	0.04	21.35 (5.46)	386	15 days
*Amphiprion barber*	65	61	0.02	11.42 (3.33)	398	18.6 days
*Chrysiptera talboti*	121	61	0.03	34.27 (5.05)	364	15.4 days*
*Halichoeres hortulanus*	108	92	0.01	7.23 (2.05)	393	32.5 days
*Pomacentrus maafu*	148	117	0.01	3.44 (1.95)	382	19.6 days

Data include the nucleotide diversity (π) and the population parameter (θ).

* *estimated from congener*.

**Table 2:** Geographic distribution of samples.

	Region:								
	West Islands		Viti Levu			Vanua Levu		Eastern Islands	
Species:	Mamanucas	Yasawas	Nananu-I-ra	Naigani	Nabukavesi	Navatu	Naigigi	Naselesele	Nacamaki
*Amblyglyphidodon orbicularis* (N = 102)	23	14	23			14	9	19	
*Amphiprion baerberi* (N = 65)	15		7	11		6	8	18	
*Chrysiptera talboti* (N = 121)	10	15	11	22	16	11	16	12	8
*Halichoeres hortulanus* (N = 108)	9	11	9	10	12	15	17	19	6
*Pomacentrus maafu* (N = 148)	21		22	19	13	40	17	16	

For all species our AMOVA analysis showed very low and non-significant levels of genetic partitioning within regions, suggesting broad levels of genetic homogeneity among species and regions. For three species (*C. talboti*, *H. hortulanus*, and *P. maafu*) pair-wise Φ_st_ values across all regional comparisons were also non-significant. In two species, *A. barberi* and *Amb. orbicularis*, there were subtle but significant indications of population differentiation (Table 3,4). For *A. barberi* we observed significant divergence between the Eastern Islands and both the Western Islands and Vanua Levu. For *Amb. orbicularis* we saw a similar divergence between the Eastern and Western Islands. None of the species showed divergence between Viti Levu and Vanua Levu suggesting that the Bligh Waters is not serving as a phylogeographic barrier for these species.

**Table 3:** [Sec s3]Results of Φ_st_ analyses for data grouped into regions. Significant values (p<.05) are in BOLD.

Species		Western Islands	Viti Levu	Vanua Levu
*Amblyglyphidodon orbicularis* (N = 102)	Western Islands (N = 37)			
	Viti Levu (N = 23)	0		
	Vanua Levu (N = 23)	0.02	0	
	Eastern Islands (N = 19)	**0.05**	0.02	0.00
*Amphiprion barberi* (N = 65)	Western Islands (N = 19)			
	Viti Levu (N = 18)	0		
	Vanua Levu (N = 14)	0	0	
	Eastern Islands (N = 14)	**0.14**	0.06	**0.09**
*Chrysiptera talboti* (N = 121)	Western Islands (N = 25)			
	Viti Levu (N = 43)	0.00		
	Vanua Levu (N = 27)	0.00	0.00	
	Eastern Islands (N = 13)	0.01	0.00	0
*Halichoeres hortulanus* (N = 108)	Western Islands (N = 11)			
	Viti Levu (N = 15)	0		
	Vanua Levu (N = 44)	0	0	
	Eastern Islands (N = 25)	0	0	0
*Pomacentrus maafu* (N = 148)	Western Islands (N = 21)			
	Viti Levu (N = 54)	0.01		
	Vanua Levu (N = 57)	0	0	
	Eastern Islands (N = 16)	0	0	0

**Table 4:** [Sec s3]Results from MIGRATE analyses.

Species		Western Islands	Viti Levu	Vanua Levu	Eastern Islands
*Amblyglyphidodon orbicularis* (N = 102)	Western Islands (N = 37)		104.5	46.5	61.5
	Viti Levu (N = 23)	241.5		61.5	100.5
	Vanua Levu (N = 23)	214.5	131.5		217.5
	Eastern Islands (N = 19)	160.5	95.5	71.5	
*Amphiprion barberi* (N = 65)	Western Islands (N = 19)		82.2	87	289.8
	Viti Levu (N = 18)	241.8		149.4	309
	Vanua Levu (N = 14)	213	162.6		273
	Eastern Islands (N = 14)	131.4	90.6	100.2	
*Chrysiptera talboti* (N = 121)	Western Islands (N = 25)		188.5	414.5	671.5
	Viti Levu (N = 43)	393.5		360.5	620.5
	Vanua Levu (N = 27)	412.5	157.5		693.5
	Eastern Islands (N = 13)	460.5	283.5	471.5	
*Halichoeres hortulanus* (N = 108)	Western Islands (N = 11)		215.4	229.8	247.8
	Viti Levu (N = 15)	310.2		213	274.2
	Vanua Levu (N = 44)	468.8	169.8		229.8
	Eastern Islands (N = 25)	451.8	168.6	142.2	
*Pomacentrus maafu* (N = 148)	Western Islands (N = 21)		180.6	403.8	159
	Viti Levu (N = 54)	330.6		437.4	441
	Vanua Levu (N = 57)	210.6	127.8		139.8
	Eastern Islands (N = 16)	393	357	411	

Values represent the median number of recruits per generation exchanged. Migration is from the column to the row. See Supporting Information S1 for full confidence intervals.

MIGRATE analysis indicated that there was no universal pattern of migration among all the species and regions (Table 4, Supporting Information S1). For those species that did not show any evidence of genetic differentiation among regions (*H. hortulanus*, *C. talboti* and *P. maafu*) migration rates predictably showed a fairly consistent pattern of the Western Islands importing far more migrants than they export, while for species showing population differentiation, migration rates were substantially lower but showed no consistent pattern.

It is important to note that migration estimates can be influenced by demographic expansion, wherein sequence similarity, which could be ascribed to high levels of migration, actually is due to shallow coalescent times. To test for a signal of recent demographic expansion we calculated Tajima's D. Two species (*C. talboti* and *P. maafu*) showed significantly negative values of Tajima's D, while a third, *H. hortulanus* had a nearly significant negative Tajima's D (p. = 0.052), Significant and negative values of Tajima's D indicate a greater number of low frequency mutations than would be expected under a neutral model of sequence evolution, suggesting a recent population expansion or selective sweep which could artificially inflate migration estimates. As expected, species with significant pair-wise differentiation (*Amb. orbicularis* and *A. barberi*) showed low levels of migration among regions and non-significant values of the Tajima's D, indicating a stable demographic history (Supporting Information S1).

Results from the isolation by distance analysis indicated two patterns occurring within the Fijian localities. Three of the four species (*C. talboti*, *H. hortulanus* and *P. maafu*) had non-significant, flat or negative correlations between geographic and genetic distances (all p<0.48), while *Amb. orbicularis* exhibited significant isolation by distance, with an r^2^ of .71 (p≪.01).

## Discussion

### Gene flow within the Fijian Archipelago

Our results indicate on the relatively small spatial scales of the Fijian Archipelago (maximum pair wise shore line distance = <400 km), genetic analysis can detect subtle but statistically significant barriers to population connectivity for some, but not all, common reef fish species. Tellingly, there was only moderate overlap among the phylogeographic patterns, with three species (*C. talboti*, *H. hortulanus* and *P. maafu*) having no structure throughout the islands. Two species (*A. barberi* and *Amb. orbicularis*) showed several instances of small but significant subdivision (Φ_st_ values ranging from 0.05 to 0.14). Migration estimates among all species were asymmetrical with no consistent pattern emerging among the species.

While there are complex and idiosyncratic patterns of subdivision among the five species in this study, the lack of differentiation between Viti Levu and Vanua Levu for all species and no consistent pattern of migration from east to west as would be mediated by the mean flow of the Bligh Waters, suggest that the Blight Waters are not acting as a major phylogeographic barrier within Fiji for these species. This is in contrast to other fast flowing ocean currents such as the Halmahera eddy [48] the Indonesian throughflow [49] and the Mona passage [50].

Two species show moderate levels of genetic subdivision. One, *Amb. orbicularis*, showed subdivision only on the largest scale, between the Eastern and Western Islands (Φ_st_ = 0.05 p<0.05), which, in conjunction with the evidence of Isolation by Distance (r^2^ = 0.71 p≪0.01), implies that the majority of gene flow occurs between spatially proximate locations [9], [51]. *Amphiprion barberi* also showed a similar Eastern vs. Western Islands pattern (Φ_st_ = 0.14 p<0.05), but additionally expressed a small but significant differentiation between the proximal Eastern Islands and Vanua Levu (Φ_st_ = 0.09 p<0.05). The MIGRATE analysis for both of these species showed limited and asymmetrical migration among all sampled areas within Fiji. With *Amb. orbicularis* the majority of the exchange being between proximal locations, while in *A. barberi* almost twice as many migrants originating in the East Islands and settling in the Western Islands than the reverse.

Both *A. barberi*, *Amb. orbicularis* are Southwest Pacific endemics and may represent cases of peripheral isolation from their more broadly distributed Indo-Pacific congeners [41]. The genus *Amphiprion* has several examples of limited dispersal [52]–[54] and our results from *A. barberi* echo these previous results. Similarly, *Amb. orbicularis* is part of a recent radiation in the Southwest pacific in which each archipelago within the region (Fiji, Tonga and Samoa) houses an endemic species of *Amblyglyphidodon* suggesting limited dispersal among regions within the Southwest Pacific [39]. Strong patterns of regional isolation may suggest limited larval dispersal capabilities that contribute to the higher levels of genetic structure on the scale of the Fijian Archipelago.

In contrast, three species, *C. talboti*, *H. hortulanus* and *P. maafu*, show no significant barriers to gene flow across the Fijian Archipelago, indicating that migration between localities is sufficient to genetically homogenize populations. However, migration estimates can be strongly influenced by population expansion, and the recovery of significantly negative Tajima's D values suggests that *C. talboti* and *P. maafu* may be experience non-equilibrium dynamics. Therefore, we focus more on the general magnitude of migration rather than the specific values. Taken together, our results mirror a number of previous studies of population connectivity in coral reef fish, which have found no significant barriers to dispersal at the within archipelago scale [25], [26], [55], [56] (but see [57], [58]).

Several recent papers have criticized mtDNA based studies [59]–[61] for multiple reasons, and have highlighted that coalescent estimates such as MIGRATE are improved with the addition of multiple loci [62]. However, a recent review by Karl and colleagues, convincingly argue that multilocus data are not always better and mtDNA is not necessarily flawed [63]. The control region is rapidly evolving [∼10% myr-1 64] providing resolution across the spatial and temporal scales herein. It is likely that additional work including increasing the spatial sampling (i.e. southern Viti Levu, Kadavu or southern islands in the Lau group) and using microsatellite markers could provide greater geographic coverage and independent molecular verifications of our hypotheses. However, the value of comparative phylogeography as envisioned by Avise [65], [66] is the power of multiple species comparisons, where multiple species serve as independent tests of regional environmental and geological processes. Comparisons of all five species highlight that they do not exhibit the same levels of differentiation, a result that successfully addresses the hypothesis at hand.

### Integrating multi-taxa data into marine reserve design

There has been a trend in conservation studies to focus on a few key surrogate species, either because their presence is indicative of a particular habitat type [67], they are frequent targets of fisheries pressure [68], or they serve as umbrella species, wherein the conservation of one species affords protection to a larger suite of species [69], [70]. Traditionally this approach has been selected to simplify data collection and allow conservation planners to refine their efforts on a suite of selected species. However this method of conservation planning has been critiqued for numerous reasons including lack of requisite life history information [71] variations in the umbrella species' habitat [71] and taxonomic biases in coverage [72]. Here, by focusing on a suite of species we gain a better understanding of the evolutionary dynamics of the system as a whole, and by extension the ability to better scale conservation measures [73].

Our findings demonstrate that the intricacies of population connectivity amongst reef fish cannot simply be generalized based on the behavior of an individual exemplar species. While we have several instances of genetic subdivision within our data set, there is only modest similarity among these patterns. Selecting a reserve design based on any single species would not adequately represent the evolutionary and ecological dynamics expressed in the other four. For example, a reserve system designed on *C. talboti H. hortulanus* or *P. maafu* would not capture the differentiation between the East and West Islands, while one based on *A. barberi* would establish a system that risks incurring opportunity costs for ‘over protecting’. Finally, while a reserve based exclusively on *Amb. orbicularis* would capture the major break within Fiji doing so would fail to draw a distinction between a species-specific result and one based on replicate findings. There is no guarantee that a reserve predicated on *Amb. orbicularis*' genetic structure would provide adequate representation in other South West Pacific reef systems and to suggest that it is a surrogate species for other countries could be dangerously myopic.

To adequately capture the dynamics of the system results from multiple species are required. Since genetic data has a high instance of type 2 errors, (failing to detect an effect when one is there), evidence of barriers to gene flow should be considered highly robust [74]. Moreover, when we do see concordance in these significant findings across multiple taxa we can make stronger inferences about the underlying evolutionary, geophysical or oceanographic dynamics of the system [75].

With this study we are moving away from single taxa paradigm towards a multi-taxa, ecosystem based approach. A transition that has occurred in other areas of terrestrial conservation biology [2], [76], and with reductions in computational and financial expenses, is emerging in marine conservation genetics [50], [73], [77], [78].We are not advocating for all conservation planning measures to include massive multi-taxa initiatives, as that is unrealistic given the restrictions on resources (financial, human or otherwise) that most researchers face. Rather, we anticipate a transition towards more taxonomically inclusive studies as in-country capacity increases and sequencing costs decrease.

## Conclusions

Previous multi-taxa studies of reef organisms have been useful in elucidating concurrent patterns of gene flow in marine systems. These estimates have ranged from limited genetic structure [26] to strong differentiation [79], occasionally within the same study [73], [77]. Our results fall in between these two extremes and demonstrate small but significant barriers to genetic exchange amongst some species within the Fijian archipelago.

These results provide sufficient justification to manage Fijian reefs at a regional scale. Ideally we would suggest a network of marine reserves within each of these regions that would operate in parallel, providing resilience and diversity within the system [80]. At minimum, we suggest that there should be at two management regions, one on the eastern and one on the western side of the country, which would encapsulate a genetic break observed in two of the five of the species sampled in this study. Ideally we suggest a network of marine reserves within each of these regions that would operate in parallel, providing resilience and diversity within the system [80]. While there is clear indication of substantial migration between regions in some species, the fact that others demonstrate genetic barriers indicates that this is not a completely open system. In particular, the migration estimates for *C. talboti* and *P. maafu* may be influenced by non-equilibrium population dynamics. If this is the case we may be overestimating connectivity among the regions. If so this argues strongly for a more finely partitioned protected area system. A precautionary approach towards reserve management will help ensure the conservation of Fiji's coral reefs [32].

Our work highlights the importance of incorporating more nuanced views of comparative phylogeography when delineating conservation programs. By incorporating data from multiple species we have shown how our views of what an ‘optimal’ reserve system change. While this study focused on a system within the South West Pacific, we believe that this work will be applicable to researchers working in other systems. Reef are threatened throughout the world [81] and there is a critical need for innovative conservation strategies to protect the vivid splendor of these ecosystems.

## Supporting Information

Supporting Information S1 (PDF)
