# Supplementary material for: Comparative Phylogeography in Fijian Coral Reef Fishes: A Multi-Taxa Approach towards Marine Reserve Design
Source: PLoS One. 2012 Oct 30;7(10):e47710. doi: 10.1371/journal.pone.0047710 (PMC3484158; doi:10.1371/journal.pone.0047710)
Supplement: Supporting Information S1 — (PDF) [file pone.0047710.s001.pdf]

Species:	Migration pattern:	2.50%	25.00%	Mode	75.00%	97.50%	Median	Mean
Amblyglyphidodon orbicularis	Eastern Islands->Vanua Levu	0	0	52.5	217	612	217.5	250.8
	Eastern Islands->Viti Levu	0	0	0.5	100	419	100.5	146
	Eastern Islands->Western Islands	0	0	0.5	61	376	61.5	113.9
	Vanua Levu->Eastern Islands	0	0	0.5	71	470	71.5	123.8
	Vanua Levu->Viti Levu	0	0	0.5	61	369	61.5	107
	Vanua Levu->Western Islands	0	0	5.5	46	183	46.5	63.9
	Viti Levu->Eastern Islands	0	0	0.5	95	473	95.5	149.9
	Viti Levu->Vanua Levu	0	0	0.5	131	514	131.5	177.8
	Viti Levu->Western Islands	0	0	0.5	104	462	104.5	152.5
	Western Islands->Eastern Islands	0	0	34.5	160	511	160.5	198.3
	Western Islands->Vanua Levu	0	39	103.5	249	608	214.5	250.3
	Western Islands->Viti Levu	0	0	23.5	114	636	241.5	266.6
Amphiprion barberi	Eastern Islands->Vanua Levu	0	0	0.6	272.4	789.6	273	316.7
	Eastern Islands->Viti Levu	0	57.6	208.2	354	843.6	309	352.7
	Eastern Islands->Western Islands	0	0	0.6	87.6	770.4	289.8	322.1
	Vanua Levu->Eastern Islands	0	0	0.6	99.6	379.2	100.2	134.6
	Vanua Levu->Viti Levu	0	0	0.6	148.8	880.8	149.4	251.3
	Vanua Levu->Western Islands	0	0	0.6	86.4	560.4	87	156.3
	Viti Levu->Eastern Islands	0	0	0.6	90	420	90.6	136.9
	Viti Levu->Vanua Levu	0	0	0.6	162	894	162.6	266.6
	Viti Levu->Western Islands	0	0	0.6	81.6	640.8	82.2	162
	Western Islands->Eastern Islands	0	0	35.4	130.8	604.8	131.4	194.1
	Western Islands->Vanua Levu	0	0	0.6	212.4	868.8	213	294.2
	Western Islands->Viti Levu	0	0	0.6	241.2	952.8	241.8	329.5
Chrysiptera talboti	Eastern Islands->Vanua Levu	223	686	980.5	994	1000	693.5	637.1
	Eastern Islands->Viti Levu	151	514	674.5	875	1000	620.5	597
	Eastern Islands->Western Islands	236	662	962.5	992	1000	671.5	619.6
	Vanua Levu->Eastern Islands	0	127	278.5	590	937	471.5	482.1
	Vanua Levu->Viti Levu	0	0	89.5	360	899	360.5	403.5
	Vanua Levu->Western Islands	0	7	167.5	422	788	414.5	446.8
	Viti Levu->Eastern Islands	0	0	0.5	283	871	283.5	344.9
	Viti Levu->Vanua Levu	0	0	0.5	157	722	157.5	231.6
	Viti Levu->Western Islands	0	0	0.5	188	749	188.5	257.7
	Western Islands->Eastern Islands	0	41	224.5	499	925	460.5	474.2
	Western Islands->Vanua Levu	0	0	0.5	411	663	412.5	446.6
	Western Islands->Viti Levu	0	51	150.5	439	904	393.5	426.7
Halichoeres hortulanus	Eastern Islands -> Vanua Levu	0	0	0.6	192	846	192.6	227.4
	Eastern Islands -> Viti Levu	0	0	0.6	273.6	975.6	274.2	276.5
	Eastern Islands -> Western Islands	0	0	29.4	247.2	862.8	247.8	470.4
	Vanua Levu -> Eastern Islands	0	0	0.6	141.6	759.6	142.2	280
	Vanua Levu -> Viti Levu	0	0	21	212.4	937.2	213	260
	Vanua Levu -> Western Islands	0	0	0.6	229.2	854.4	229.8	474.3
	Viti Levu -> Eastern Islands	0	0	0.6	168	907.2	168.6	360.3
	Viti Levu -> Vanua Levu	0	0	29.4	169.2	811.2	169.8	311.9
	Viti Levu -> Western Islands	0	0	0.6	214.8	802.8	215.4	368.1
	Western Islands -> Eastern Islands	0	0	0.6	184.8	1032	451.8	319
	Western Islands -> Vanua Levu	0	339.6	517.8	662.4	980.4	468.6	305.2
	Western Islands -> Viti Levu	0	0	0.6	309.6	943.2	310.2	283.9
Pomacentrus maafu	Eastern Islands -> Vanua Levu	0	0	0.6	139.2	963.6	139.8	290.1
	Eastern Islands -> Viti Levu	0	0	0.6	404.4	1086	441	476.9
	Eastern Islands -> Western Islands	0	0	0.6	158.4	866.4	159	255.2
	Vanua Levu -> Eastern Islands	0	0	31.8	409.2	1053.6	411	458.2
	Vanua Levu -> Viti Levu	0	0	0.6	144	1045.2	437.4	459.3
	Vanua Levu -> Western Islands	0	0	0.6	184.8	316.8	403.8	469
	Viti Levu -> Eastern Islands	0	0	0.6	356.4	1052.4	357	429.3
	Viti Levu -> Vanua Levu	0	0	0.6	127.2	861.6	127.8	249.4
	Viti Levu -> Western Islands	0	0	0.6	180	1017.6	180.6	328.4
	Western Islands -> Eastern Islands	0	0	0.6	392.4	1023.6	393	437.4
	Western Islands -> Vanua Levu	0	0	18.6	210	954	210.6	328.9
	Western Islands -> Viti Levu	0	0	0.6	302.4	1014	330.6	387.5
